# Distinguishing Electron Diffusion and Extraction in Methylammonium Lead Iodide

**DOI:** 10.1021/acs.jpclett.3c00082

**Published:** 2023-03-21

**Authors:** P. E. Brown, A. Ruseckas, L. K. Jagadamma, O. Blaszczyk, J. R. Harwell, N. Mica, E. Zysman-Colman, I. D. W. Samuel

**Affiliations:** † Organic Semiconductor Centre, SUPA, School of Physics and Astronomy, University of St Andrews, North Haugh, St Andrews, Fife KY16 9SS, United Kingdom; ‡ Organic Semiconductor Centre, EaStCHEM, School of Chemistry, University of St Andrews, North Haugh, St Andrews, Fife KY16 9ST, United Kingdom

## Abstract

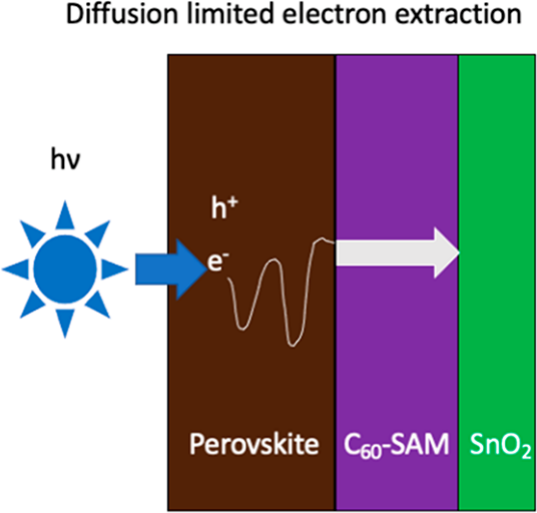

Charge diffusion and extraction are crucial steps in the operation of solar cells. Here we show that time-resolved photoluminescence can be used to study electron diffusion in hybrid perovskite films and subsequent transfer to the adjacent electron extraction layer. As diffusion and transfer to the extraction layer are consecutive processes, they can be hard to distinguish, but by exciting from each side of the sample we can separate them and identify which process limits charge extraction. We find that the introduction of a fullerene monolayer between the methylammonium lead iodide (MAPbI_3_) and the electron-transporting SnO_2_ layers greatly increases the electron transfer velocity between them to the extent that electron diffusion limits the rate of electron extraction. Our results suggest that increasing the electron diffusion coefficient in MAPbI_3_ would further enhance the electron extraction rate, which could result in more efficient n–i–p type solar cells.

Hybrid perovskite solar cells represent an exciting field of research as the materials combine desirable properties such as large absorption coefficients,^1^ high radiative efficiencies,^2^ and long charge carrier diffusion lengths^3−5^ with simple processing from solution.^6,7^ There has been spectacular growth in the power conversion efficiency (PCE) of perovskite solar cells over the last 10 years, achieving to date >25% PCE.^8^ An important part of this progress is improvement in charge extraction efficiency.^9,10^ Charge extraction has to overcome recombination of charge carriers in the absorber layer and at the interfaces with extraction layers after one type of charge carrier is transferred to a charge extraction layer.^11,12^ Rapid charge extraction helps prevent lingering charge carriers that can cause damage to the absorption or extraction layers, thereby improving the device stability.^13,14^

A highly efficient electron extraction layer in n–i–p perovskite solar cell devices has been SnO_2_, with close to 21% PCE reported for these devices.^15−17^ Recent studies have shown that increased PCE and stability of the devices are produced by incorporating fullerene interlayers between the hybrid perovskite and SnO_2_ layers.^18−20^ This has so far been explained by better energy level alignment for the extraction of electrons^21,22^ and a reduction of the number of charge recombination events at the interface between the absorber and extraction layers.^23−26^ However, carrier diffusion and transfer to the extraction layer are consecutive processes and therefore can be hard to distinguish.

In this Letter we use time-resolved photoluminescence (TRPL) measurements with excitation from different sides of the film to investigate electron extraction (Figure 1). The signal detected in the TRPL measurement is produced by radiative recombination of photogenerated electrons and holes and is quenched when carriers are extracted. When illuminating from the perovskite side, charge carriers are primarily generated far from the electron extraction layer and must diffuse through the perovskite to it, providing information on charge diffusion kinetics through the perovskite layer. When illuminating from the substrate side, charge carriers are primarily generated near the electron extraction layer where there is less of a distance for the carriers to diffuse to reach the perovskite–extraction layer interface; thus, information on the electron transfer velocity to the extraction layer can be obtained. We previously used this method to study hole diffusion and extraction from MAPbI_3_ to a hole extraction layer of either NiO or PEDOT:PSS.^27^ We showed that a hole diffusion coefficient of 2.2 cm^2^ s^–1^ in MAPbI_3_ films, which was independent of carrier density, indicates band-like hole transport. Here, we study electron diffusion and extraction to electron extraction layers and determine the electron diffusion coefficient to be approximately 70 times smaller than the value for holes in films made by the same preparation procedure.^27^

**Figure 1:**
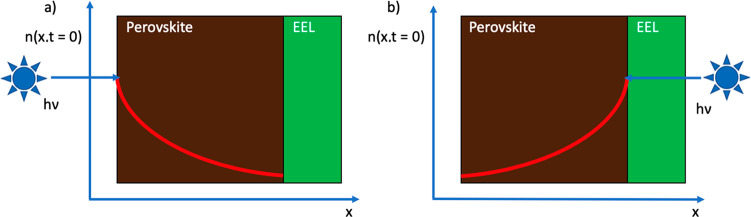
Initial electron and hole density profiles immediately after an ultrashort excitation pulse at 640 nm from (a) perovskite and (b) electron extraction layer side illumination. *n* represents electron density, *x* represents depth into the perovskite film, *t* represents time after initial excitation, and EEL represents the electron extraction layer.

The perfect quencher boundary condition used in many studies assumes a charge carrier density of zero at the quencher interface, i.e., an infinite rate of quenching at the interface. Quenching is then limited by diffusion. This assumption usually works well for materials with slow diffusion of charge carriers and fast interfacial transfer velocity such as organic semiconductors.^28^ Charge carriers in perovskites, however, have higher diffusion coefficients, and so this assumption can lead to inaccurate modeling. Real quenchers have a finite rate of quenching, and so there can be a flux of charge carriers between the interface and the bulk, especially at high charge carrier concentrations. This can result in an underestimated diffusion coefficient as the perfect quencher model does not consider that some charges can reach the quenching interface and then diffuse back into the perovskite bulk instead of being quenched. For these situations it is important to modify the boundary conditions for the quenching interface, such as those taking into account the finite electron transfer velocity (*S*_T_) to the electron extraction layer.^29^ This is referred to as an imperfect quencher model. We model the data, taking into account the absorption profile through the sample, and determine the electron diffusion coefficient (*D*) and *S*_T_. This gives a detailed mechanistic representation of charge extraction that allows us to determine which components in the architecture are limiting charge extraction and so can be targeted for improvement.

The thickness of the MAPbI_3_ layers was 400 nm, which is comparable to that used in optimized devices.^30^Figure 2 shows typical absorbance and photoluminescence (PL) spectra of the MAPbI_3_ films used in this study, which align with previous reports.^31,32^ The identical PL maxima at 770 nm in different extraction layers evidence a consistent bandgap (Figure S1). Different excitation wavelengths were explored to provide shallow or deep penetration of excitation light into MAPbI_3_. They returned very similar PL spectra, indicating that the material has a uniform bandgap throughout the film (Figure S2). Consistency of the crystal structure and grain size for MAPbI_3_ spin-coated onto different extraction layers was also demonstrated through X-ray diffractograms and scanning electron microscopy images (Figures S3 and S4).

**Figure 2:**
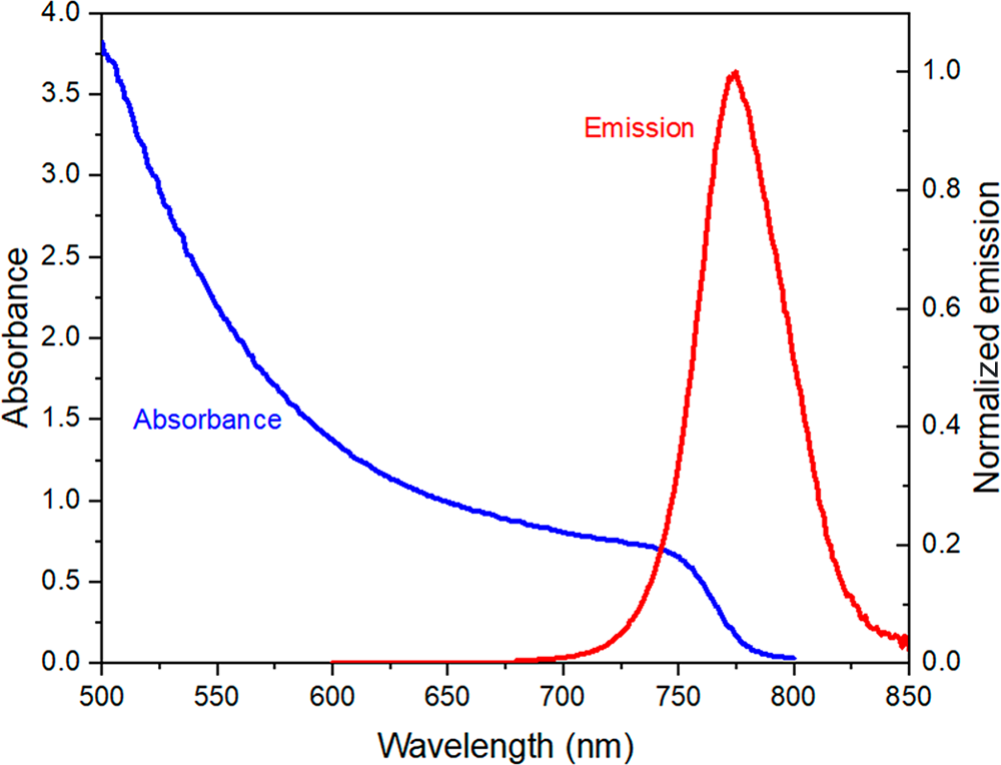
Absorbance and steady-state photoluminescence (excited at 590 nm) spectra for a MAPbI_3_ sample of thickness 400 nm.

We measured TRPL using an excitation wavelength of 640 nm. This ensured that ∼90% of the incident photons were absorbed to achieve the distinct profiles of the initial carrier density when exciting from different sides of the film. The PL was detected at 770 nm. Excitation from each side in bare MAPbI_3_ films gave identical PL decays (Figure S5), whereas MAPbI_3_ films on SnO_2_ showed faster decays than the bare MAPbI_3_ film, indicating dynamic PL quenching by the electron extraction layer (Figure 3). Figure 3 also shows that excitation from the SnO_2_ side leads to faster PL decay than excitation from the MAPbI_3_ side. As explained in Figure 1, this happens because in the former case charges are generated closer to the SnO_2_ layer. The reference film is important to use as this accounts for effects such as nonradiative and reabsorption processes so that we can compare against our film with EEL to investigate electron extraction effects. To estimate the electron extraction efficiency, we first determined the rate constant of electron extraction by taking the ratio of the PL decay at the extraction layer to that of the bare MAPbI_3_ (Figure S6a). The kinetics of the PL ratio represent the additional decay by electron extraction with other decay processes in the bare film accounted for. Validation of this approach has been previously presented.^33^ This gave a rate constant of 0.02 ns^–1^ for extraction to SnO_2_. Then we divided this by the PL decay rate of the sample with extraction layer to obtain the extraction efficiency of 44%. This rate constant is comparable to that of other studies with this extraction layer.^18^

**Figure 3:**
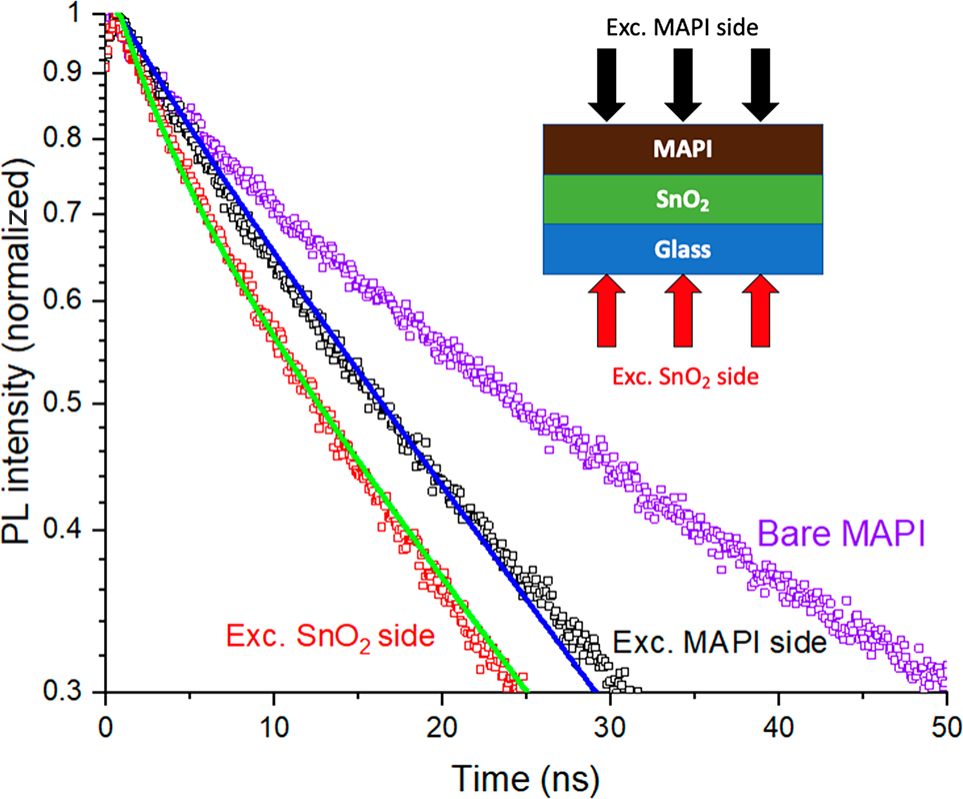
PL decays on a logarithmic scale of bare MAPbI_3_ film on glass and of MAPbI_3_ on a SnO_2_ electron extraction layer when measured with excitation from two different sides. Excitation was at 640 nm. Solid lines show fits to an imperfect quencher model obtaining values of *D* = 0.033 cm^2^ s^–1^ and *S*_T_ = 57 m s^–1^. The diagram shows the two illumination scenarios.

Photoluminescence in semiconductors, such as MAPbI_3_, occurs by band-to-band recombination of free electrons and holes. Time-resolved PL intensity is proportional to the product of the electron density in the conduction band *n*(*x*, *t*) and the hole density in the valence band *p*(*x*, *t*) integrated over the thickness, *L*, of the MAPbI_3_ layer.^34^
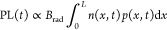
1where *B*_rad_ is the radiative recombination rate constant. We performed measurements that detect emission from the same side as the excitation (reflection geometry), which minimize any potential reabsorption and emission processes. In general, the local electron density can be described by the rate equation
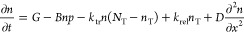
2where *G* is the charge generation rate; *B* is the rate constant of bimolecular recombination; *k*_tr_ and *k*_rel_ are the rate constants of electron trapping and release, respectively; *N*_T_ is the total density of electron traps in the MAPbI_3_ film; *n*_T_ is the density of trapped electrons; and *D* is the electron diffusion coefficient. Since the excitation pulse duration of 0.3 ns is much shorter than the PL decay, the generation term in eq 2 can be omitted. We found that PL decays showed only a weak dependence on excitation density below 500 nJ cm^–2^, indicating that the bimolecular recombination term (*Bnp*) is negligible at this excitation density. Only mobile carriers can recombine radiatively; therefore, the PL decay in bare MAPbI_3_ films can be understood as being dominated by electron trapping, which slows down with time when more trap states are filled.^35,36^ Partial release of carriers from the traps would have a similar effect on the free carrier density as trap filling; therefore, we combine trapping and release terms into a single time-dependent decay rate, *k*_bare_, to describe the decay of free electron density. Thus
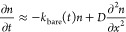
3

About 90% of excitation light at 640 nm is absorbed in the MAPbI_3_ films, so the contribution of the back reflection to excitation is negligible, and the initial electron density profile throughout the film is determined by the Beer–Lambert law
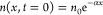
4where *n*_0_ is the initial electron density and α is the absorption coefficient at the excitation wavelength. We model our results with mobile holes recombining with trapped electrons. Because of the fast hole diffusion in MAPbI_3_, we assume that the hole trapping is negligible and the holes quickly become evenly distributed throughout the film. This is a fair assumption based on our previous work with hole extraction.^27^ As the density of mobile holes is much larger than that of mobile electrons, the loss of hole density to bimolecular recombination is very small. Therefore, we assume that the hole density *p*(*t*) is constant throughout electron extraction time.

The PL decay of the bare MAPbI_3_ film was fitted with a biexponential decay function to represent the time-dependent PL decay rate in the bare MAPbI_3_ film. This is a parametrization to best represent the shape of the decay curve. We then explored whether a perfect quencher could account for our measurements, using the boundary conditions for the quenching and reflecting interfaces expressed by
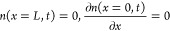
5where x=0 is the surface of the perovskite film and x=L is the perovskite/EEL interface. In the perfect quencher model, there is only one parameter that can be optimized to obtain an accurate fit: the electron diffusion coefficient. However, there is a large misfit between what the model predicts and what the data suggest. This can be seen by the large difference in lifetimes between the perovskite side and SnO_2_ side models when compared with the data obtained by exciting from each side (Figure S7). The misfit is likely due to the electron extraction layer not acting to quickly extract all of the incoming charge carriers.

As we were unable to account for these results with a perfect quencher model, we instead explored a model with finite quenching at the interface. This involved assuming that the diffusive flux of charge carriers at the interface is equal to the quenching rate, leading to the boundary condition in eq 6. The model was applied to the TRPL data in Figure 3. The model has two fitting parameters: the diffusion coefficient and the transfer velocity. The addition of the transfer velocity takes into account the finite quenching rate at the interface and enables us to probe how effectively different interfaces quench.
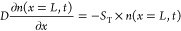
6

Matching the curves as well as matching the difference in lifetime between the MAPbI_3_ side and SnO_2_ side decay profiles, we can learn about both charge diffusion and the rate of charge transfer at the interface. The best fit was obtained with the following fitting parameters: *D* = 0.033 ± 0.004 cm^2^ s^–1^ and *S*_T_ = 57 ± 4 m s^–1^ (Figure 3). We also measured PL kinetics with about 10 ps time resolution using a streak camera and a similar energy density as in TCSPC measurements. We observed slightly faster PL decay on the EEL as compared to the bare film within the 2 ns time window but only for excitation from the EEL side (Figure S8). The streak camera data can be fitted satisfactorily with the same *D* and *S*_T_ parameters as TCSPC. This informs us that we capture all relevant trapping and extraction processes with TCSPC. Further information on the effects of changing fitting parameters can be found in Figures S9 and S10. The much better fit employing a finite transfer velocity shows that for an unfunctionalized SnO_2_ electron extraction layer, both the interface and charge diffusion play a role in electron extraction. The best fits were determined by having the lowest χ^2^ value between the PL data and fit. Using the fitted parameters and a typical PL lifetime (τ) value of 36 ns, the 1-D diffusion length (*L*_D_) was calculated to be 0.49 μm using eq 7, which is shorter than the diffusion length for holes due to electrons having a lower diffusion coefficient than holes.^27^ We were most interested in the 1-D diffusion length perpendicular to the extraction layer interface because this is the direction most relevant for charge extraction in a photovoltaic device. The actual *L*_D_ may be larger because our model does not account for electrons that are released from traps on a longer time scale.
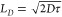
7

The ability to determine the transfer velocity enables the comparison of charge extraction when using different charge extraction layers. We explored this by applying a fullerene self-assembled monolayer (C_60_-SAM) to the SnO_2_ layer. The C_60_-SAM is chemically bonded to the SnO_2_ layer surface via a carboxylic acid anchoring group. This introduces new states at the SnO_2_–MAPbI_3_ interface while having minimal effect on the bulk properties of the MAPbI_3_ film.^21^ The result is shown in Figure 4 where the TRPL signal decays faster than the similar sample without the C_60_-SAM (Figure 3). We estimated a rate constant of 0.05 ns^–1^ for electron extraction to the layer functionalized by the C_60_-SAM, which gives an extraction efficiency of 68% (Figure S6b). This rate constant is comparable to other studies with this extraction layer.^18,37^

**Figure 4:**
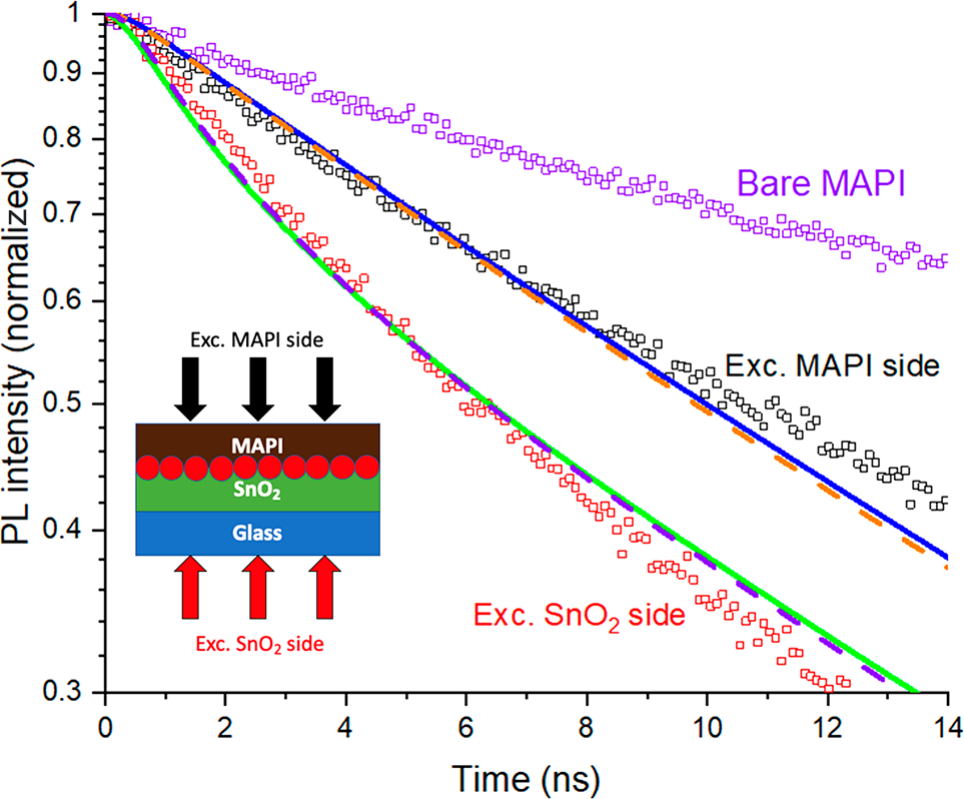
PL decays on a logarithmic scale of bare MAPI and of MAPI on a SnO_2_ with C_60_-SAM electron extraction layer when measured with excitation from two different sides. Excitation was at 640 nm. Dashed lines show fitting using a perfect quencher model with *D* = 0.034 cm^2^ s^–1^. Solid lines show fits to an imperfect quencher model obtaining values of *D* = 0.034 cm^2^ s^–1^ and *S*_T_ = 1100 m s^–1^. The diagram shows the two illumination scenarios. Red circles represent C_60_-SAM.

The imperfect quencher model fitting to the PL decays gave an electron diffusion coefficient of 0.034 cm^2^ s^–1^, which is similar to that of unfunctionalized SnO_2_, while the transfer velocity was increased from 57 m s^–1^ to about 1100 m s^–1^. The simulated fit (solid line) shows faster decay than the data at times *t* > 10 ns for excitation from the MAPbI_3_ side and slower for excitation from the C_60_-SAM side. This can be understood to be a result of fast electron trapping and slow release, which slows electron diffusion through the MAPbI_3_ layer with time. In our model we fit with a time-independent *D* and so focus on a fast extraction component by fitting the first 15 ns of the PL decay to mimic the situation in working cells where the internal electric field speeds up extraction by adding a carrier drift component and faster release. For comparison, we also modeled these data with a perfect quencher assumption (dashed line). We obtained a fit very similar to that of the imperfect quencher model. This is because the transfer velocity is sufficiently high that the extraction layer effectively acts as a perfect quencher.

An important consideration when fitting multiple parameters is the possibility that they may be correlated. To consider this possibility, the data were fitted with various combinations of *D* and *S*_T_ parameters, and the resulting χ^2^ values for MAPbI_3_ on SnO_2_ are plotted in Figure 5. The lowest χ^2^ values can be found along the slice in Figure 5a corresponding to an *S*_T_/*D* ratio of approximately 1700 m cm^–2^ (using the units of the figure). However, there is a clear minimum value along this slice of the graph at *D* = 0.033 ± 0.004 cm^2^ s^–1^, as can be seen in Figure S11. This shape of the graph indicates that operation is in the interface-limited regime where a faster diffusion requires a higher transfer velocity in order to achieve a good fit. This can be thought of as a higher diffusion coefficient increases the probability of electrons to diffuse away from the extraction layer, so a higher transfer velocity is required to counteract electron escape from the interface and optimize overall electron extraction. From this we can see that SnO_2_ as an electron extraction layer without passivation layers will limit device efficiency as the electron transfer to the extraction layer cannot keep up with the electron diffusion away from the interface.

**Figure 5:**
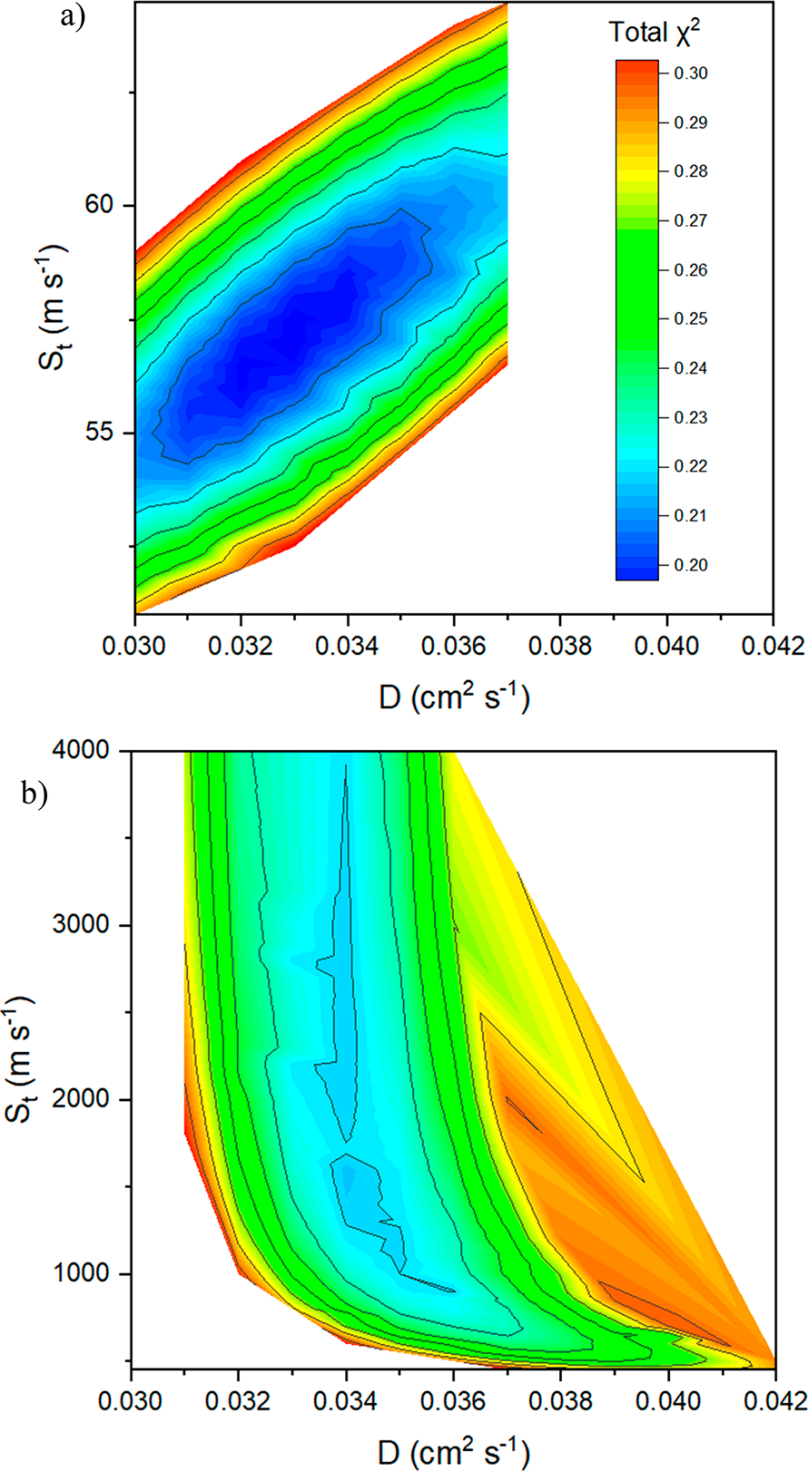
Contour plot representing the χ^2^ parameter associated with adjusting the two fitting parameters of *D* and *S*_T_ when using (a) a SnO_2_ electron extraction layer or (b) a SnO_2_ + C_60_-SAM electron extraction layer.

The contour plot for the SnO_2_ + C_60_ SAM electron extraction layer has a very different shape (Figure 5b). For the range of diffusion coefficient values between 0.032 and 0.036 cm^2^ s^–1^, there is a clear region where increasing the transfer velocity has little-to-no effect on the goodness of the fit. There is a clear minimum value of χ^2^ at *D* = 0.034 cm^2^ s^–1^, as can be seen in Figure S12. This indicates that the electron extraction is entirely diffusion-limited as any improvements in transfer velocity will have essentially no effect on extraction time.

The obtained value of *D* = 0.033 cm^2^ s^–1^ for electrons is lower than our measurement for holes of *D* = 2.2 cm^2^ s^-1^ by a factor of approximately 70. This could arise from electron diffusion being limited by fast trapping and slow release from shallow traps near the conduction band. Electrons are still extracted but at much slower rates than holes due to a higher density of traps for electrons than for holes. A previous study by time-resolved microwave conductivity also found a much lower electron mobility than hole mobility.^38^ Li et al. reported higher electron diffusion coefficient in the range of 0.06–0.18 cm^2^ s^–1^ in MAPbI_3_ films using a technique similar to ours but with a PCBM electron extraction layer.^39^ This suggests that their samples had a lower density of electron traps than ours, or perhaps PCBM may have partially diffused into the perovskite film due to no chemisorption to the extraction layer. Several groups have measured an ambipolar diffusion coefficient (*D*_a_) in the range of 0.28–0.4 cm^2^ s^–1^ at a high carrier density of >10^17^ cm^–3^ using the transient grating technique (eq 8).^40,41^*D*_a_ can be calculated with
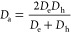
8where *D*_e_ is the electron diffusion coefficient and *D*_h_ is the hole diffusion coefficient. Using our reported value of *D*_h_, this gives *D*_a_ = 0.065 cm^2^ s^–1^ in our samples, which were measured at an average carrier density of about 4 × 10^16^ cm^–3^. The higher excitation density used in transient grating measurements and a limited time range of a few nanoseconds likely resulted in more filled electron traps, which can explain the higher *D*_a_ values in transient grating measurements.

The large enhancement in *S*_T_ with chemisorption of the fullerene monolayer on SnO_2_ leads to electron extraction being limited by diffusion. Our results suggest that trap passivation in MAPbI_3_ is needed to increase diffusion coefficient to enable faster electron extraction, which would lead to more efficient solar cells. This emphasizes the importance of charge generation and charge transport layers for optimal device performance.

Time-resolved photoluminescence with two-sided illumination has successfully been applied to study electron diffusion in MAPbI_3_ and extraction to the electron extraction layer. We observed different PL decay times as a result of illumination from different sides of the MAPbI_3_ film and so can distinguish between electron diffusion in the perovskite absorber layer and electron transfer to the electron extraction layer. We found that incorporating a fullerene monolayer between MAPbI_3_ and SnO_2_ significantly increases the electron extraction rate, so that it becomes limited by electron diffusion. The electron diffusion coefficient in MAPbI_3_ of 0.033 cm^2^ s^–1^ is much lower than the hole diffusion coefficient of 2.2 cm^2^ s^–1^, suggesting that electron traps substantially limit electron transport and extraction rate. Further improvements in the electron extraction rate will require enhancement of electron diffusion in the charge generation layer.

## Experimental Methods

Samples were prepared within a nitrogen-filled glovebox as described in the Supporting Information and transferred into a nitrogen-filled chamber for optical measurements. PL decays were recorded in reflection geometry with the time-correlated single-photon counting (TCSPC) module on a FLS980 fluorimeter from Edinburgh Instruments using 0.3 ns light pulses at 640 nm for excitation with a pulse energy density of about 500 nJ/cm^2^ and a pulse repetition rate of 200 kHz (Figure S13). PL kinetics in the 2 ns time window were measured with about 10 ps resolution using a Hamamatsu streak camera and 200 fs light pulses for excitation with a similar energy density as in TCSPC measurements. Time-integrated photoluminescence spectra were recorded on the same fluorimeter using a xenon arc lamp and monochromator for excitation.

## Data Availability

The research data for this publication can be accessed at 10.17630/049fbc45-a235-44f3-a27f-e06061af17a4.

## Supporting Information Available

Sample preparation, photoluminescence spectra, X-ray diffraction patterns, scanning electron microscopy images, electron extraction efficiency estimation, influence of fitting parameters on fits, minimum χ^2^ analysis, and schematic of experimental setup (PDF)
