# Supplementary material for: Distinguishing Electron Diffusion and Extraction in Methylammonium Lead Iodide
Source: J Phys Chem Lett. 2023 Mar 21;14(12):3007–13. doi: 10.1021/acs.jpclett.3c00082 (PMC10068735; doi:10.1021/acs.jpclett.3c00082)
Supplement: Supplementary file 1 — jz3c00082_si_001.pdf [file jz3c00082_si_001.pdf]

Supporting Information

Distinguishing Electron Diffusion and Extraction in Methylammonium Lead Iodide

P. E. Brown,[a,b] A. Ruseckas,[a] L. K. Jagadamma,[a] O. Blaszczyk,[a] J. R. Harwell,[a] N. Mica,[a] E. Zysman-Colman[b] and I. D. W. Samuel[a]*.*

[a] *Organic Semiconductor Centre, SUPA, School of Physics and Astronomy, University of St Andrews, North Haugh, St Andrews Fife, KY16 9SS, United Kingdom*

[b] *Organic Semiconductor Centre, EaStCHEM, School of Chemistry, University of St Andrews, North Haugh, St Andrews, Fife, KY16 9ST, United Kingdom*

Table of Contents

Sample preparation S2

Photoluminescence spectra S3

X-ray diffraction S5

Scanning electron microscopy S6

Illumination side comparison of a reference sample S8

Electron extraction efficiency S9

Influence of fitting parameters on fits S10

Minimum χ^2 analysis S14

Experimental setup S16

Sample preparation

Chemicals purchased and used as received include lead iodide (TCI chemicals), methylammonium iodide (Dyesol) powders, dimethylsulfoxide (Sigma-Aldrich), dimethylformamide (Sigma-Aldrich), tetrahydrofuran (Alfa-Aesar), diethyl ether (Acros Organics) solvents and $C_{60}CO_2H$ stock solution (Lumtec). They were opened inside a nitrogen atmosphere glovebox and were stored there. A SnO_2 nanoparticle solution was opened in air and stored when not in use wrapped in parafilm in dry, dark storage.

Each glass substrate was first sonicated for 40 minutes in solutions of Hellmanex III soap solution, then sonicated followed by being rinsed in DI water three times, sonicated in acetone then isopropanol for 15 minutes before being dried with a N_2 gun and plasma ashed on the side that films were to be deposited. Each electron extraction layer was spin coated onto the glass substrate prior to spin coating the perovskite layer on top.

Thin films of transport material and perovskite were deposited on glass substrates by spin-coating. The SnO_2 film was prepared from a nanoparticle colloidal stock solution (Alfa Aesar) by diluting it in a 1:6.5 volume ratio with DI water. The mixture was then vortex mixed together to ensure a uniform dispersion. The prepared solution was then spin-coated onto the cleaned glass substrates at 3000 rpm for 30 seconds followed by annealing at 150 °C for 30 minutes. Processing was done in air ambient conditions. The thickness of the prepared SnO_2 film was ~ 30 nm.

The C_{60}-SAM solution was prepared by dissolving C_{60}-SAM powder at a concentration of 0.1 mg/ml in a 1:1 mixture of chlorobenzene and tetrahydrofuran. The substrates were dipped in this solution for 2 minutes after which excess solvent was removed with a cleanroom wipe. The substrate was then washed with 100 µL of chlorobenzene whilst spin coated at 2000 rpm for 30 seconds. All steps were carried out within a nitrogen filled glovebox.

Thin films of methyl ammonium lead iodide ($CH_3NH_3PbI_3$) (MAPI) were deposited on glass substrates with or without electron extraction layer via spin coating within a nitrogen filled glovebox. The precursor solution for producing the MAPI layer was prepared by mixing 461 mg of PbI_2 and 159 mg of methylammonium iodide in a mixed solvent of dimethyl sulfoxide (66 µL) (DMSO) and dimethyl formamide (636 µL) (DMF). The precursor solution was stirred for 1 hour at room temperature and then spin-coated onto the glass substrates at 4000 rpm for 30 seconds. Immediately after 7 seconds of the spin coating cycle, diethyl ether (700 µL) (serving as an antisolvent) was added by pipette onto the perovskite layer. Immediately after the spin coating process, the substrate was annealed at 100 °C under reduced pressure (~ 100 mbar) for 1 minute followed by 2 minutes without reduced pressure. Average film thickness was measured to be 400 nm with a Dektak profilometer. All films were transferred from the glovebox into a nitrogen-filled chamber for optical measurements.

Photoluminescence spectra

Time-integrated photoluminescence spectra were recorded with and Edinburgh instruments FLS980 fluorimeter using xenon arc lamp and monochromator for excitation.

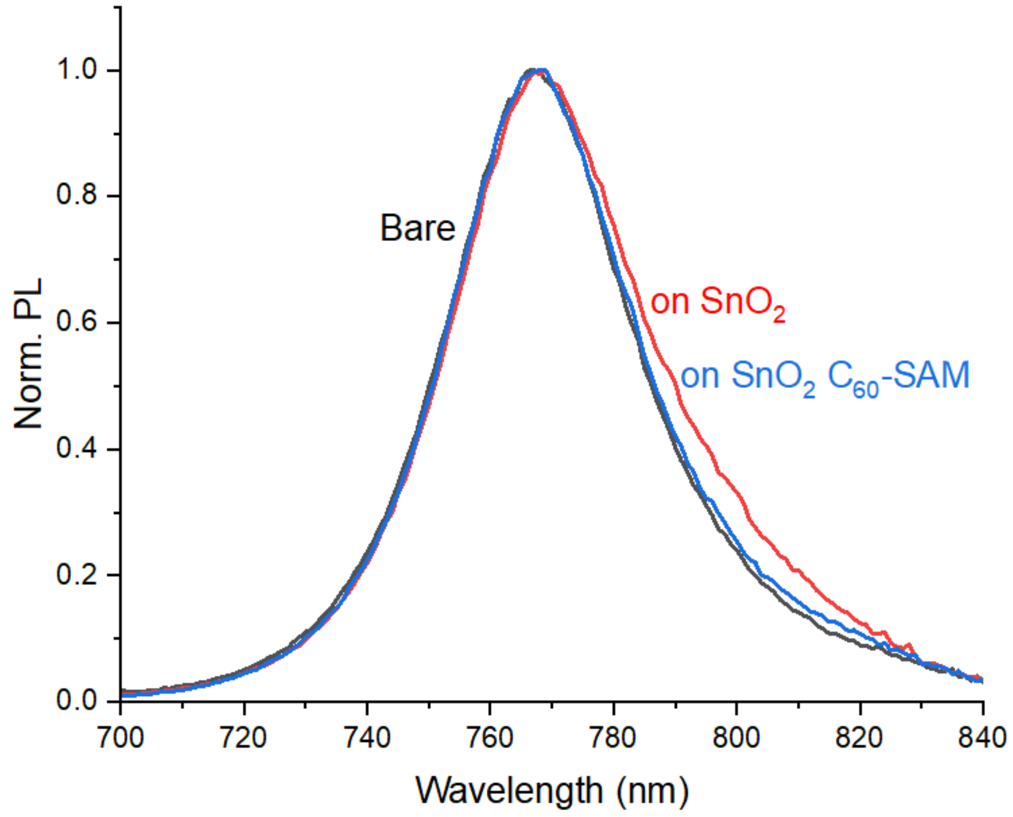

Figure S1: Normalised photoluminescence spectra of MAPI films prepared on different substrates. The black line represents a bare sample (on glass without transport layer), the red line represents the film on SnO_2 extraction layer and the blue line represents the film deposited on a C_{60}-SAM on top of SnO_2. Excitation was at 515 nm.

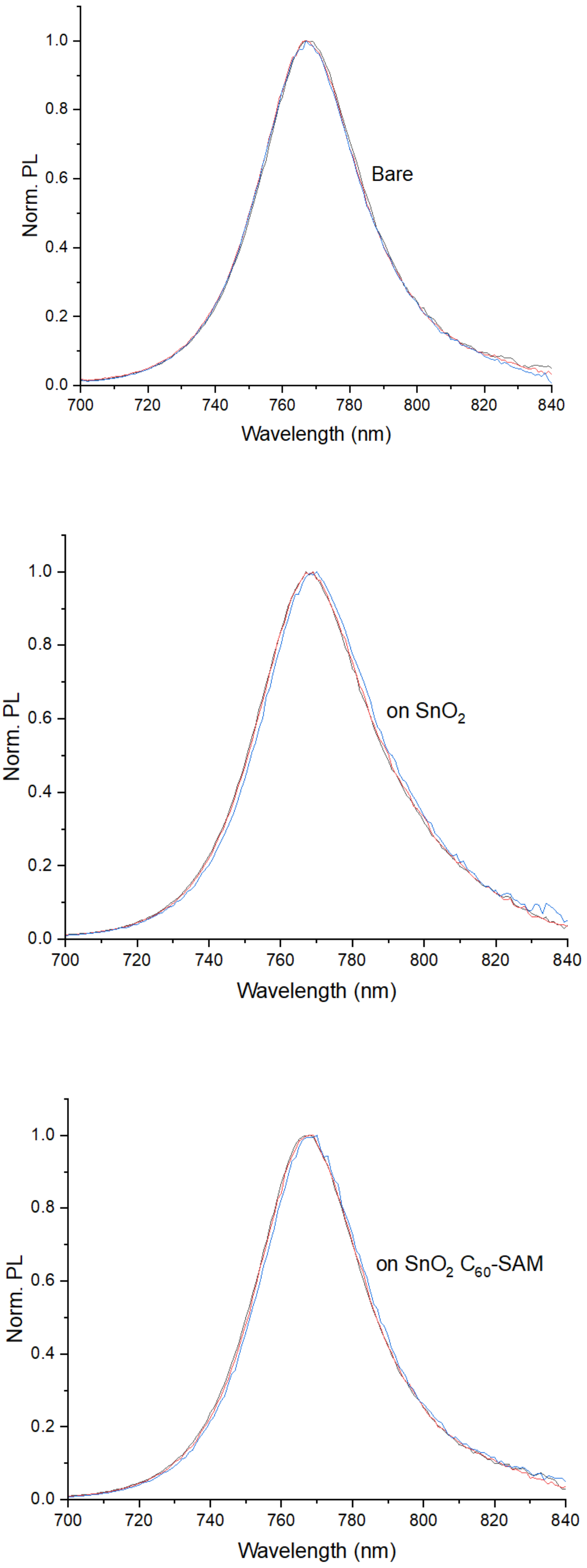

Figure S2: Normalised time-integrated photoluminescence spectra of MAPI films measured in reflection geometry using different excitation wavelengths. Black line represents 400 nm excitation, red line represents 500 nm excitation and blue line 600 nm excitation.

X-ray diffraction

X-ray diffraction measurement were performed to check for crystallinity changes when spin coating on different extraction layers (Figure S3). Position of peaks showed consistency with previous XRD measurements of MAPI.[1] There is very little shifting of peaks between extraction layers, suggesting that both the crystallinity and defect states in the bulk of the films are similar.

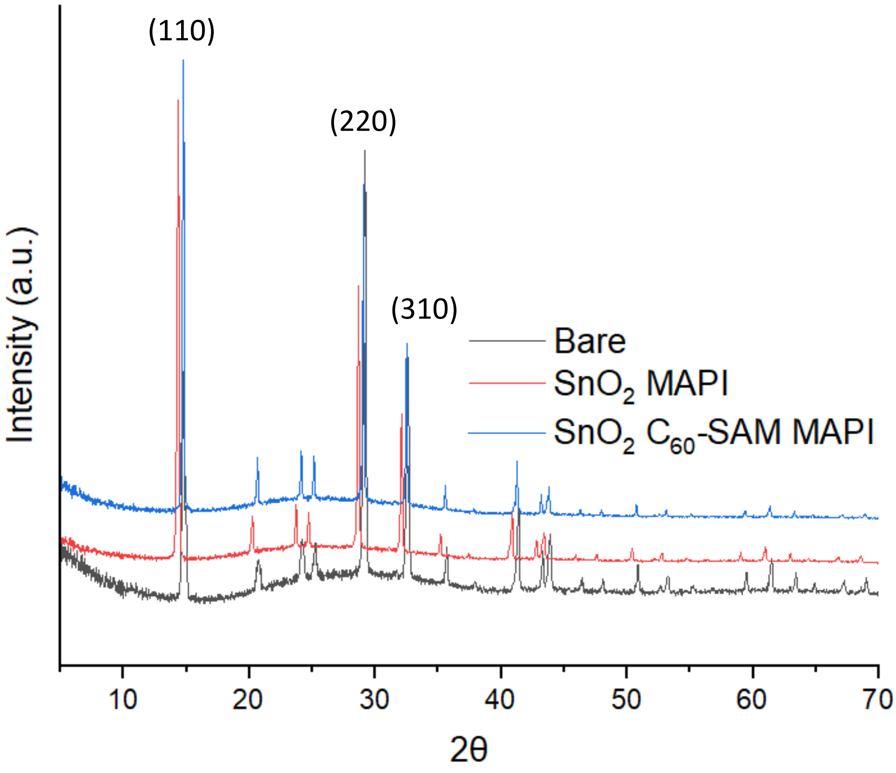

Figure S3: XRD patterns of MAPI on different extraction layers. Diffractometers are operated in reflection, Bragg Brentano,Theta -2Theta mode. Data collected at room temperature.

Scanning electron microscopy

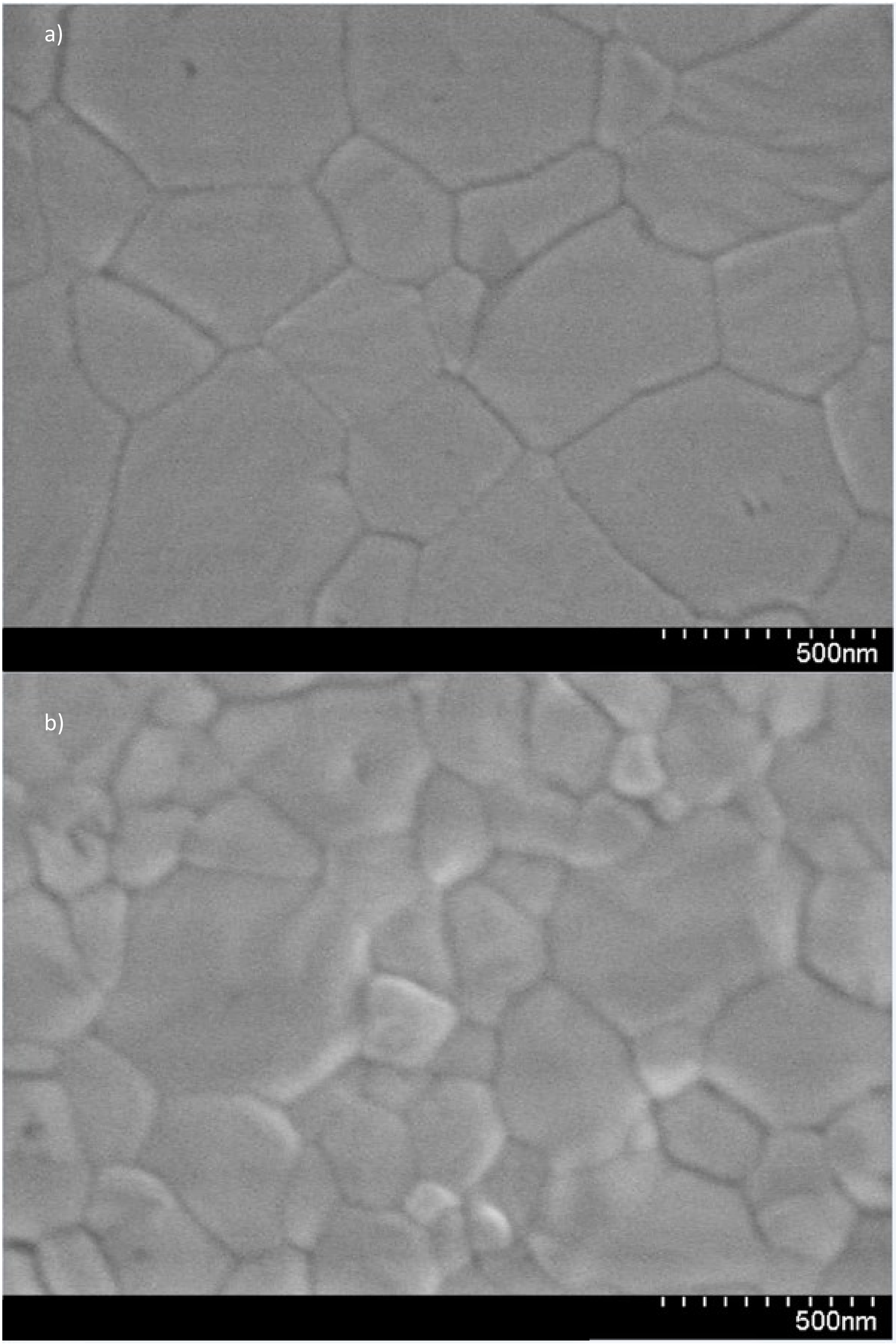

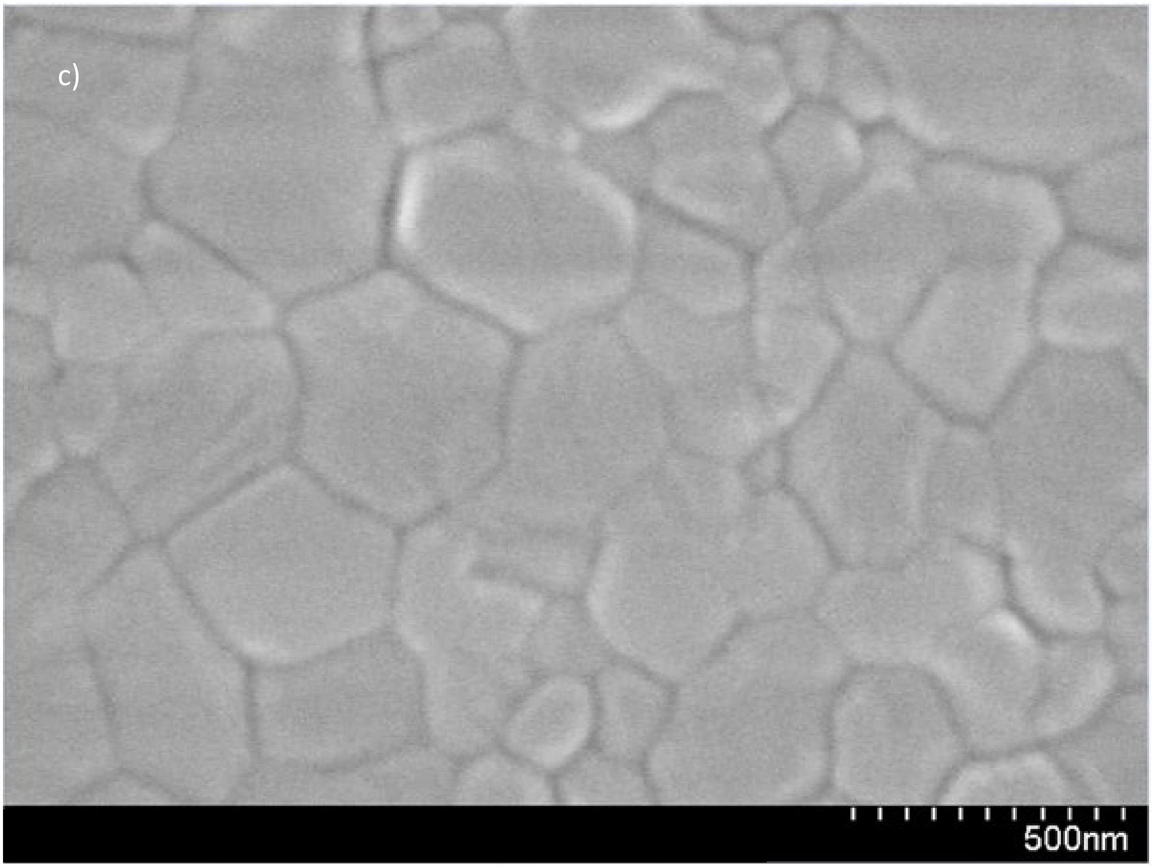

Figure S4: SEM images of a) MAPI film without extraction layer b) MAPI film with SnO_2 extraction layer c) MAPI film with SnO_2 C_{60}-SAM extraction layer. This was performed to observe the morphology of the perovskite grown on different extraction layers. Similar grain size and morphology are seen in all the samples.

Illumination side comparison of a reference sample

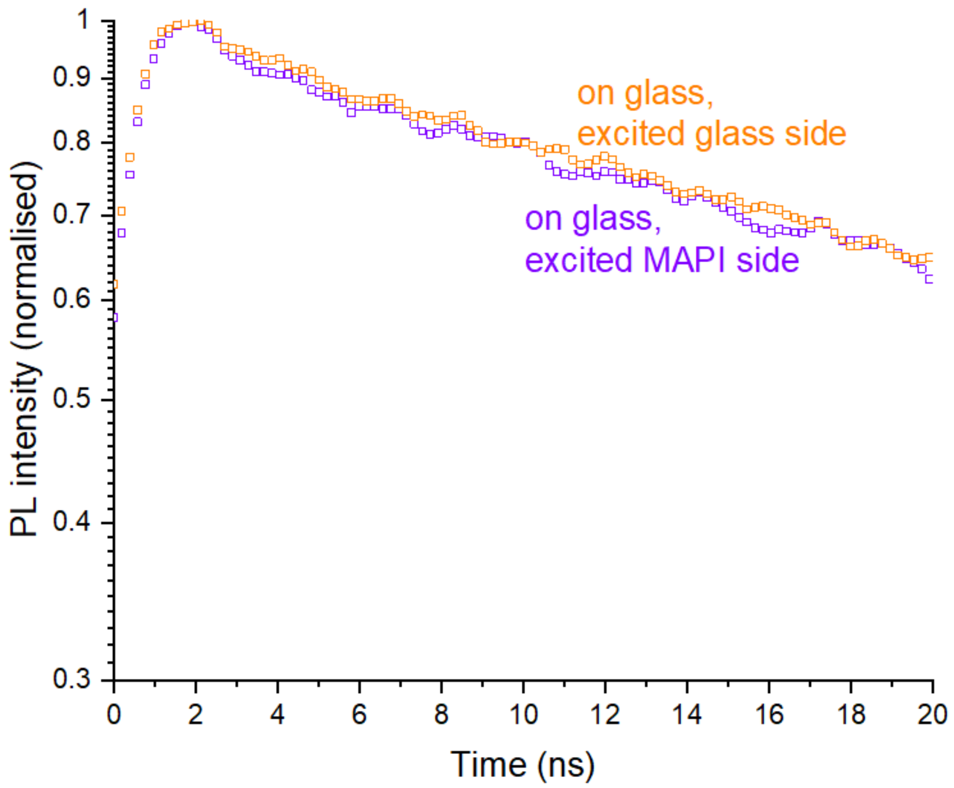

Figure S5: PL decays of bare MAPI when measured with excitation from two different sides.

Electron extraction efficiency

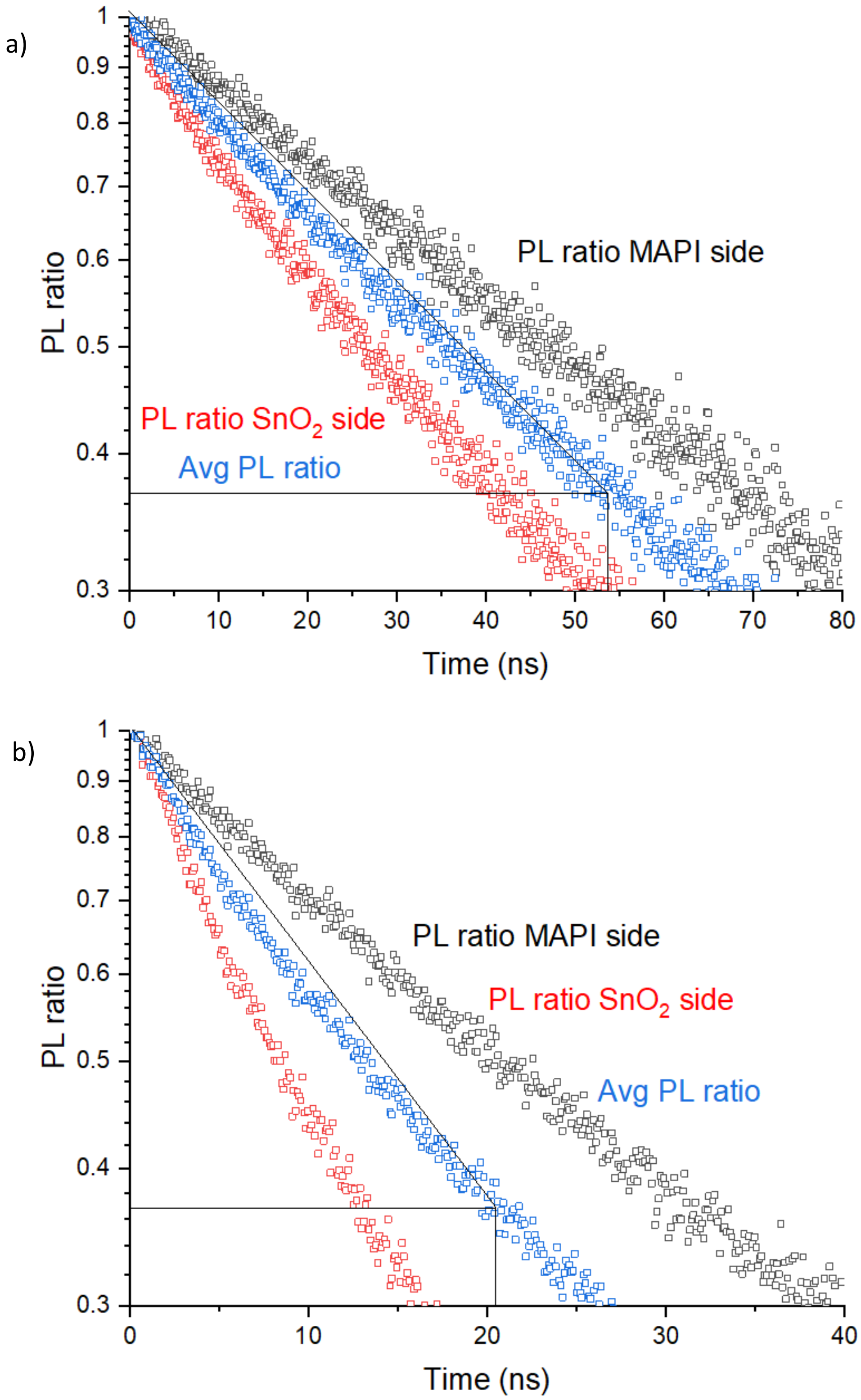

Figure S6: Ratio of PL decay on quencher to PL decay on glas for a) SnO_2 as quencher b) SnO_2 + C_{60}-SAM as quencher. Curves are labelled with the side the sample is excited from. “Avg PL ratio” represents the average of the PL ratio obtained from both sides of the sample. Lines represent the extraction time constant from the data for the average of both sides.

Influence of fitting parameters on fits

The fits to PL decays obtained with time-correlated single photon counting (TCSPC) on the SnO_2 electron extraction layer using perfect quencher and imperfect quencher models (Figure S7). The perfect quencher assumption gives much larger difference between the two-side excitation indicating it is not suitable for this sample. In contrast, much better fits are obtained for the imperfect quencher assumption.

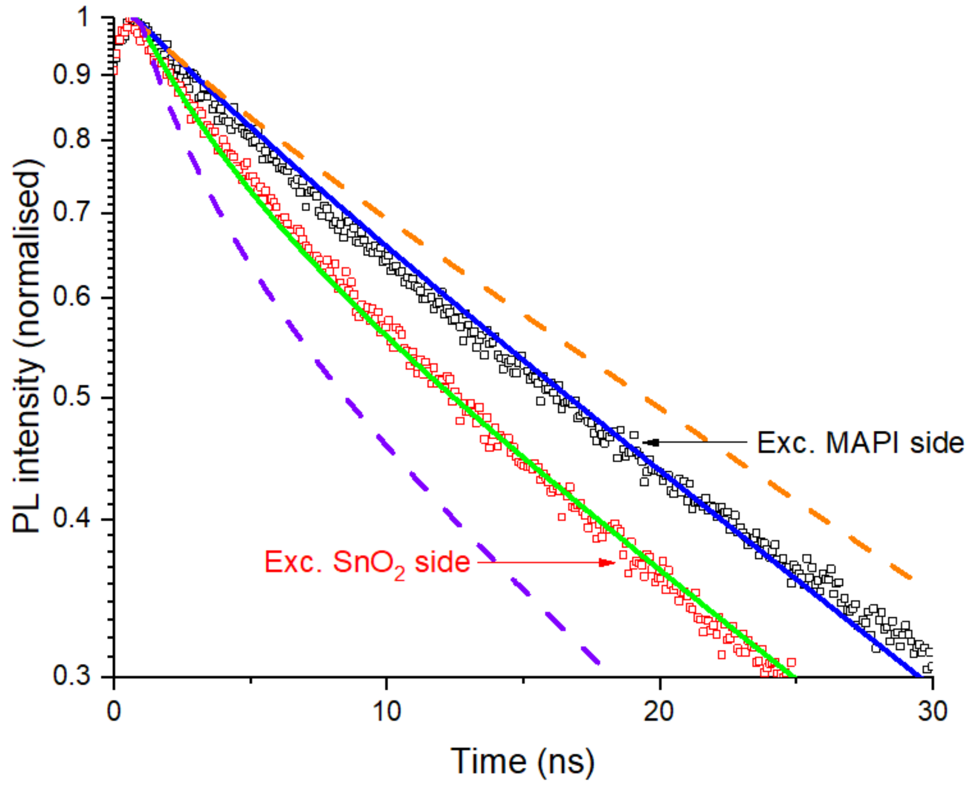

Figure S7: PL decays in MAPI on a SnO_2 electron extraction layer when measured with excitation from two different sides using TCSPC with an excitation pulse wavelength of 640 nm. Solid lines show imperfect quencher model fitting with D=0.033 $cm^2\ s^{-1}$ and S_t=57 m s^{-1}. Dashed lines show fitting using a perfect quencher model with D=0.02 $cm^2\ s^{-1}$.

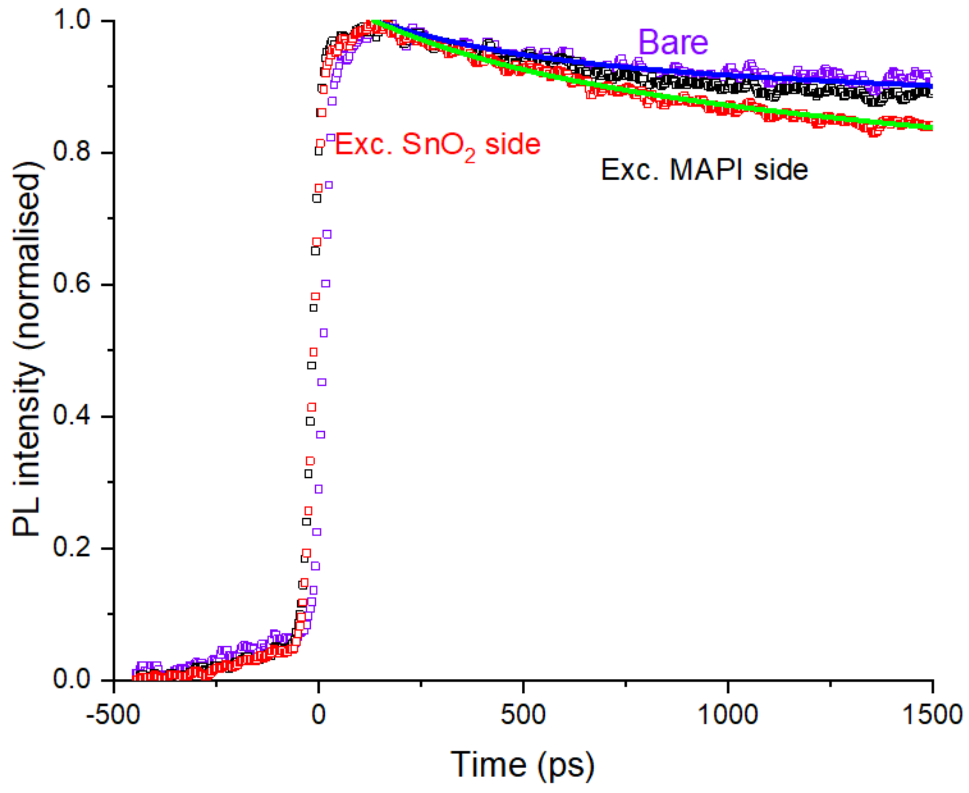

Figure S8: PL kinetics on a short timescale in bare MAPI and with a SnO_2 electron extraction layer measured with excitation from two different sides at 640 nm. Solid lines show imperfect quencher model fitting with D=0.033 cm^2 s^{-1} and S_T=57 m s^{-1}. PL decays in the 2 ns time window were measured with about 10 ps resolution using Hamamatsu streak camera and 200 fs light pulses for excitation with a similar energy density as in TCSPC measurements.

We have also explored how changing parameters affects our fits. Increasing the diffusion coefficient in the fit, reduces the difference in lifetime between the fits of the MAPI side and SnO_2 side decay profiles (from Figure S9 solid lines to dashed lines). This is because a higher diffusion coefficient means charges can diffuse across the MAPI film faster, reducing the effect of exciting from different sides. The value of transfer velocity influences the magnitude of the lifetime that the model predicts. For example, in the case where charge extraction is interface limited such as with a SnO_2 electron extraction layer, then increasing the transfer velocity will reduce the PL lifetime of both the MAPI side and SnO_2 side simulated decays (from Figure S10 solid lines to dashed lines).

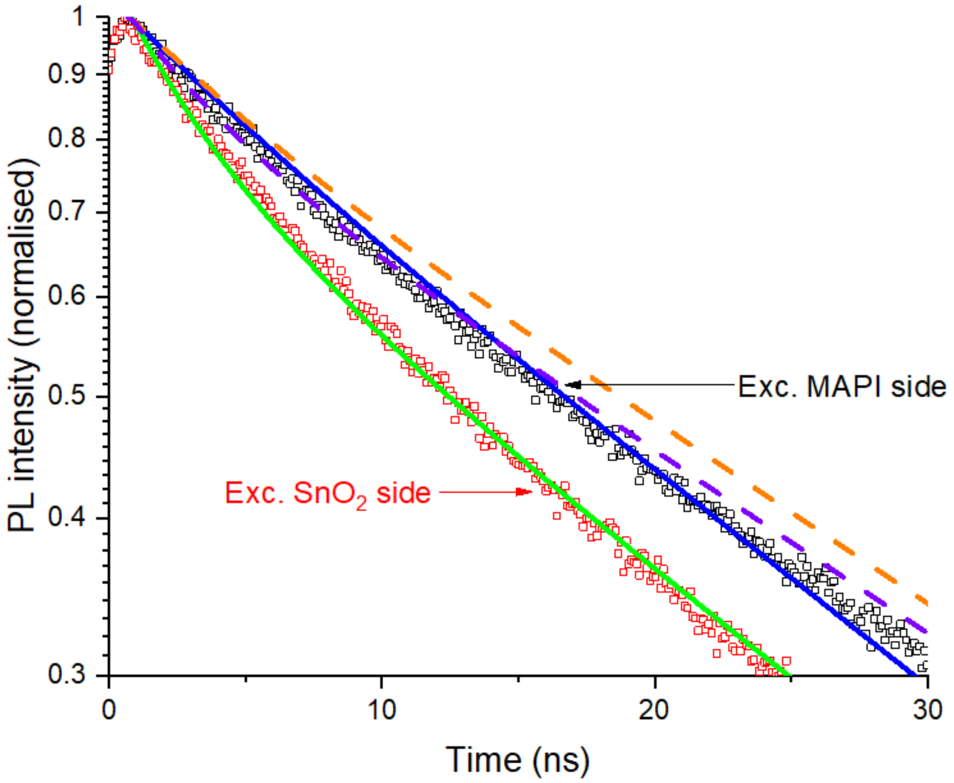

Figure S9: PL decays in MAPI on a SnO_2 electron extraction layer when measured with excitation from two different sides using TCSPC with an excitation pulse wavelength of 640 nm. Solid lines show imperfect quencher model fitting with D=0.033 $cm^2\ s^{-1}$ and S_T=57 m s^{-1}. Dashed lines show the simulated decays when increasing D by a factor of 2 from the best fit whilst S_T remains the same (D=0.066 $cm^2\ s^{-1}$ and S_T=57 m s^{-1}).

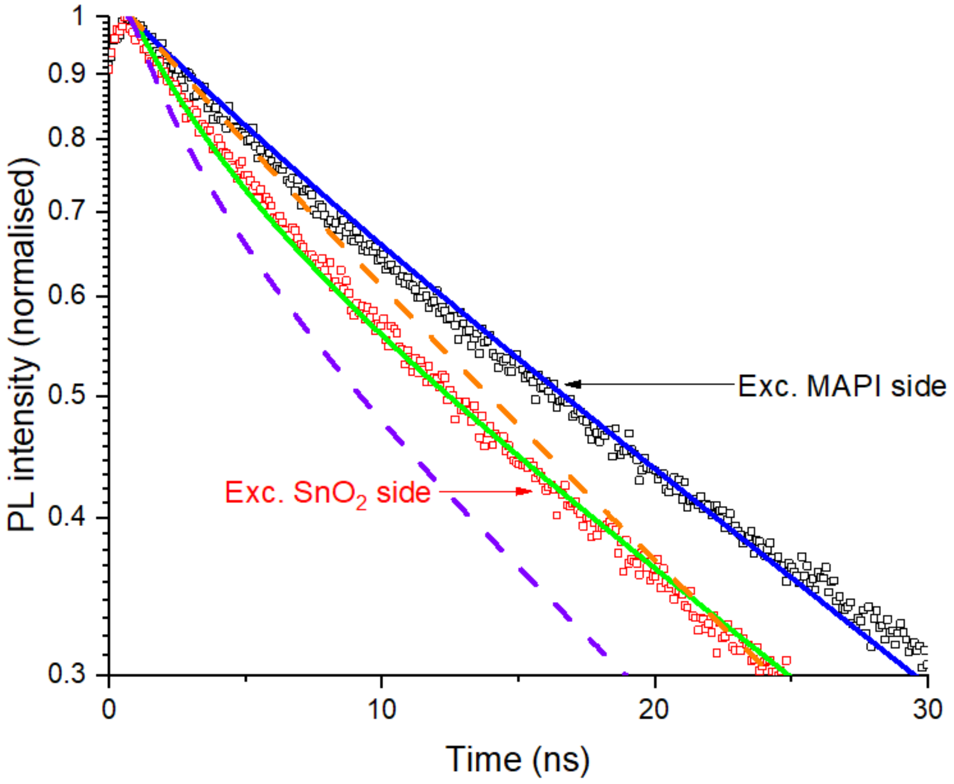

Figure S10: PL decays in MAPI on a SnO_2 electron extraction layer when measured with excitation from two different sides using TCSPC with an excitation pulse wavelength of 640 nm. Solid lines show imperfect quencher model fitting with D=0.033 cm^2 s^{-1} and S_T=57 m s^{-1}. Dashed lines show the simulated decays when increasing S_T by a factor of 2 from the best fit whilst D remains the same (D=0.033 cm^2 s^{-1} and S_T=114 m s^{-1}).

Minimum χ^2 analysis

The contour plot graphs in the main paper were further investigated to determine how well-defined the lowest χ^2 fits were and whether the modelled parameters of diffusion coefficient and transfer velocity are precisely obtained. This was seen by plotting the diffusion coefficient against the total χ^2 value for the SnO_2 electron extraction layer case (Figure S11) with S_t/D ratio of 1700 m cm^{-2} (approximately the diagonal slice through the contour plot that gives the best fits). From this there is a clear well-defined minimum χ^2 value at a diffusion coefficient of 0.034 cm^2 s^{-1} meaning that there is a precise determination of this parameter. A similar result is obtained for the SnO_2 + C_{60}-SAM (Figure S12) although the diffusion coefficient that gives the lowest χ^2 value for this graph is less well-defined. This is due to the more complex shape of the associated contour plot's minimum χ^2 values and so when plotting the lowest χ^2 for each diffusion coefficient value, an asymmetric trend is seen with a steeper slope for below D = 0.034 cm^2 s^{-1} and a shallower slope above D = 0.034 cm^2 s^{-1}.

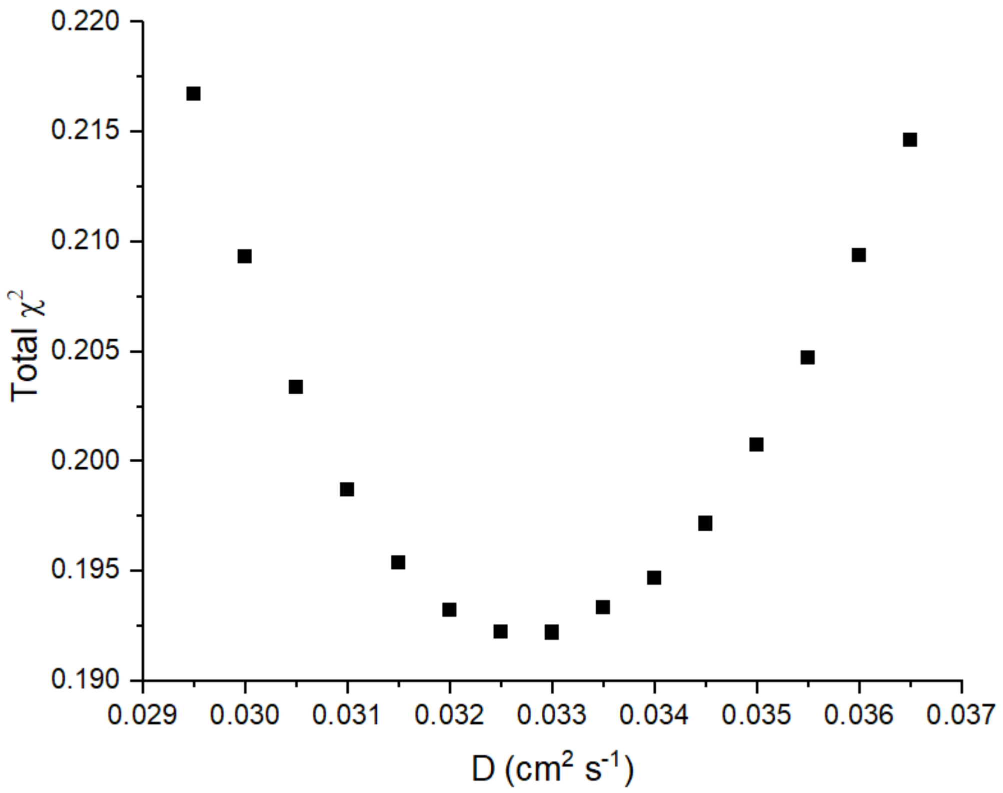

Figure S11: Plot representing the minimum χ^2 for each D value along the set S_t/D ratio of 1700 m cm^{-2}. The sample is MAPI on a SnO_2 electron extraction layer when measured with excitation from two different sides using TCSPC.

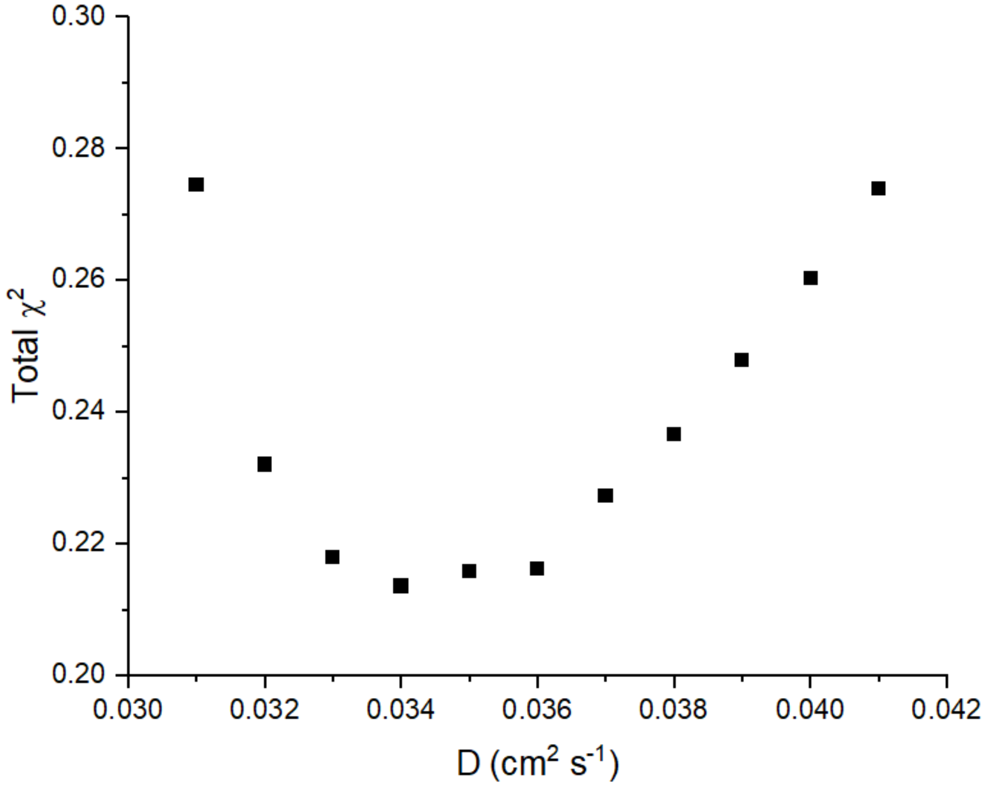

Figure S12: Plot representing the minimum χ^2 for each D value for a SnO_2 + C_{60}-SAM electron extraction layer.

Experimental setup

PL decays on a nanosecond time scale were recorded in reflection geometry with the time-correlated single photon counting module on a FLS980 fluorimeter from Edinburgh Instruments using 0.3 ns light pulses at 640 nm for excitation (Figure S13).

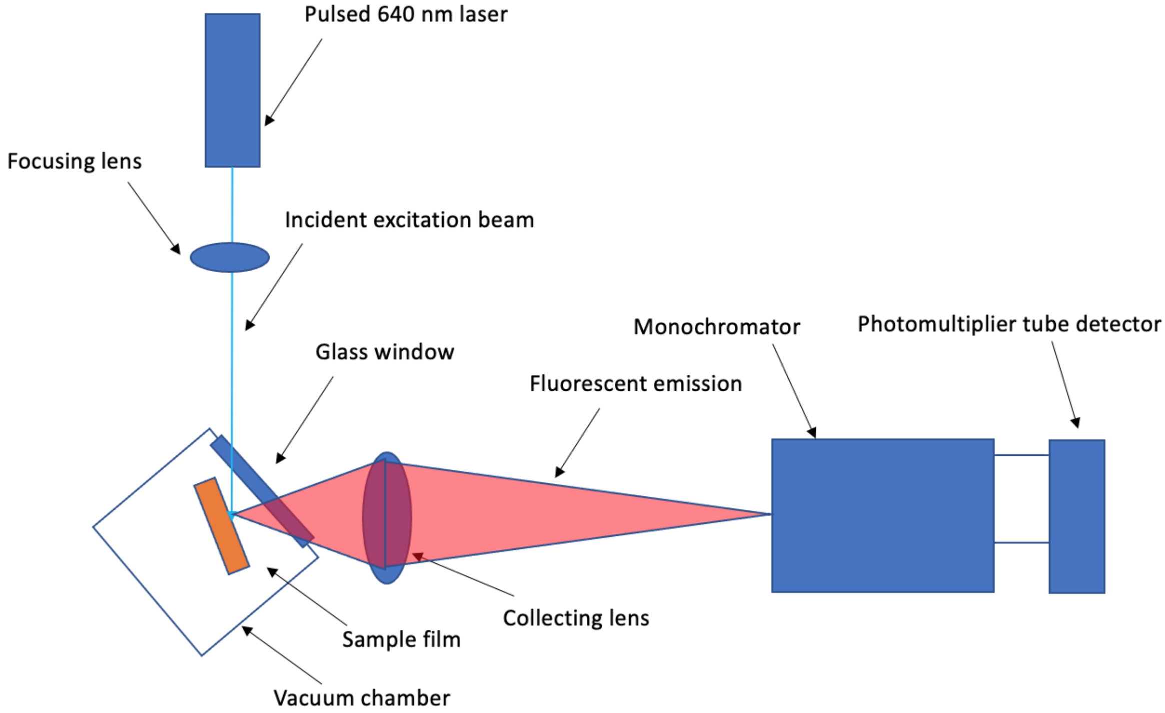

Figure S13: Schematic representing the setup used for TCSPC with an Edinburgh instruments FLS980 fluorimeter.

(1) Jagadamma, L. K.; Blaszczyk, O.; Sajjad, M. T.; Ruseckas, A.; Samuel, I. D. Efficient indoor pin hybrid perovskite solar cells using low temperature solution processed NiO as hole extraction layers. *Sol. Energy Mater. Sol. Cells* **2019**, *201*, 110071.
